# Factors associated with communicative and financial barriers to healthcare utilization among people with selected citizenships in Germany: results from a nationwide cross-sectional study

**DOI:** 10.1186/s12913-026-15932-x

**Published:** 2026-10-24

**Authors:** Lena Goerigk, Katja Kajikhina, Claudia Hövener, Carmen Koschollek

**Affiliations:** 1 Robert Koch Institute, Gerichtsstraße 27, Berlin, 13347 Germany; 2 Alice Salomon University of Applied Sciences Berlin, Berlin, Germany

**Keywords:** Migration, Utilization, Discrimination, Health inequity, Language proficiency, Socioeconomic status, Access to care

## Abstract

**Background:**

Research has shown that migrants in Germany face various barriers to healthcare access, including communicative and financial barriers. This study examined which sociodemographic, migration-related, and psychosocial factors are associated with these barriers in order to better understand lower healthcare utilization among migrants.

**Methods:**

We used data from the nationwide cross-sectional health interview survey GEDA Fokus, which included people with Croatian, Italian, Polish, Syrian, or Turkish citizenship living in Germany. Descriptive, univariable, and multivariable analyses were conducted separately for communicative and financial barriers while accounting for relevant social factors.

**Results:**

Of the 6,038 participants, 16.2% reported communicative barriers and 9.0% reported financial barriers to healthcare utilization. According to the regression analyses, gender, age, German language proficiency, and self-reported experiences of racist discrimination were associated with communicative barriers. For financial barriers, associations with gender, educational level, and experiences of discrimination due to the socioeconomic situation were found.

**Conclusion:**

The results indicate that demographic, socioeconomic, migration-related, and psychosocial factors are associated with communicative and financial barriers to healthcare utilization. Gender and experiences of discrimination were significantly associated with both communicative and financial barriers. In practice, the provision of qualified translation and interpretation services, anti-discrimination and awareness training for healthcare staff, and the expansion of low-threshold, financially accessible services could help promote more equitable access to healthcare among migrants.

**Supplementary Information:**

The online version contains supplementary material available at https://doi.org/10.1186/s12913-026-15932-x.

## Introduction

The right to the highest attainable standard of physical and mental health, as defined in the Universal Declaration of Human Rights [1], has been recognized by the signatory states, including Germany [2]. To ensure this right, equal and needs-based access to healthcare is essential. Access to and utilization of healthcare services, such as medical treatment, the provision of medicines, and health-related information, are crucial to well-being. In Germany, health insurance is mandatory, and all insured individuals have access to healthcare. The German healthcare system consists of statutory health insurance and private health insurance. Statutory health insurance covers the general population through income-based contributions and includes co-payments (e.g., for medication or hospital stays) within legal limits, while private health insurance is only accessible to certain groups within the population (e.g., high earners and the self-employed), and premiums depend on individual risk profiles and selected benefits [3]. Certain optional services outside the statutory benefits catalog (IGeL, individual health services) are generally paid out of pocket [4]. However, some individuals remain uninsured and consequently face barriers to accessing regular healthcare, resulting in limited treatment options [5].

Specific restrictions apply to the healthcare of asylum seekers, who do not have access to regular health services during the first 36 months after arrival in accordance with the Asylum Seekers Benefits Act (§§ 4 and 6); only in cases of acute illness, pain, or emergencies are they eligible to claim limited services [6]. Hence, the healthcare system is not fully accessible to all people living in Germany, e.g. for migrants. In Germany, there are different concepts used to define migrants and their descendants. Those concepts are partly based on variables such as country of birth (the person and/ or their parents), citizenship – current or at birth –, or even language-related characteristics [7]. When we speak about migrants within this paper, we more broadly refer to people with own or familial biographical connection to migration rather than one of the above-mentioned concepts – unless otherwise stated.

Research indicates that migrants and their descendants in Germany tend to utilize healthcare services at lower rates than the general population [8–15]. Various barriers, including limited knowledge of the local healthcare system, administrative requirements, language barriers, legal status, duration of residence, and experiences of discrimination, have been described as factors associated with healthcare utilization and health outcomes [2, 16]. Furthermore, people who experience racism are more likely to have lower levels of trust in healthcare systems and health professionals than those who do not report such experiences [17].

A study from Germany reported that some migrant groups may delay or avoid medical treatment because of limited language proficiency and unfamiliarity with the healthcare system, frequently relying on relatives, including children, as informal interpreters [18]. Furthermore, inadequate translation services and communication problems contribute to discrimination and misdiagnosis and hinder access to care for migrant patients in Europe [10, 19]. A European scoping review found that inadequate interpreting and cultural mediation services, a lack of health information in patients’ native languages, and communication difficulties between patients and healthcare providers are major obstacles to accessing healthcare. These barriers occur throughout the entire treatment process, from initial contact with the healthcare system to consultations, and often lead to delays in care or patients foregoing necessary healthcare altogether [20].

Direct expenditures, such as out-of-pocket payments, as well as indirect healthcare costs, including travel expenses, lost wages, and childcare, can affect healthcare utilization and pose barriers for people with limited financial resources [12, 21, 22]. Even in a healthcare system with broad insurance coverage, such as Germany’s statutory health insurance system, these costs can influence access to care. Individuals with lower incomes often have to prioritize essential living expenses such as rent, food, and childcare, which can make managing healthcare costs particularly difficult. In Germany, the average income of migrants is lower, and the proportion of people at risk of poverty is higher among this group than among the native-born population [23]. As a result, migrants may face financial barriers that delay or hinder their utilization of healthcare services [24].

Given the importance of communicative and financial barriers to healthcare utilization, the aim of this paper is to examine which demographic, socioeconomic, migration-related, and psychosocial factors are associated with these barriers and thus hinder access to healthcare. Identifying factors associated with such barriers is important for better understanding lower rates of healthcare utilization among migrants and their descendants (hereafter referred to as “migrants” for simplicity, while acknowledging that not all migrant groups belong to ethnic minorities and that not all racialized or ethnic communities are migrants). This may help inform efforts to address disparities in health outcomes, such as a higher prevalence of chronic diseases [25, 26] and lower health-related quality of life [15].

## Methods

### Study design

Data from the multilingual, multimodal health interview survey “Gesundheit in Deutschland aktuell: Fokus” (GEDA Fokus; German Health Update: Fokus) (November 2021–May 2022) were used for the present statistical analyses. This study was conducted by the Robert Koch Institute (RKI) with a specific focus on adults aged 18–79 years with selected citizenships (Croatian, Italian, Polish, Syrian, or Turkish) residing in Germany. The citizenship groups were selected on the basis of model calculations described in detail elsewhere [27]. The sample was randomly drawn from residents’ registration offices in 99 cities and municipalities throughout Germany based on first, second, or third citizenship (thus including dual citizenship) [27].

Data were collected using a sequential mixed-mode design with bilingual study materials and questionnaires (e.g., German–Italian for people with Italian citizenship). In addition to an initially offered web-based questionnaire, participants were later offered the option of completing a bilingual paper-based questionnaire. In larger cities, participants who had not responded to previous invitations were also offered the opportunity to participate in a personal or telephone interview, conducted partly by bilingual interviewers [27]. It was aimed to include 1,200 participants per citizenship group. Further details as well as a detailed description of the study population stratified by citizenship are provided in the study protocol, where also a detailed overview of the topics surveyed can be found [27].

A total of 6,038 people participated in the GEDA Fokus survey. The corresponding response rate was 18.4% (response rate 1 according to the standards of the American Association for Public Opinion Research) [27, 28].

### Indicators

#### Communicative barriers in healthcare utilization

Following previous research [29] this variable was composed of two items: “In the last 12 months, have you needed an examination or treatment but did not make use of it? (1) ...because you did not know who to turn to? (2) ...because you would have needed translation/language mediation but this was not offered?” Response options were “Yes,” “No,” and “No need for examination or treatment.” The variable was dichotomized for analysis. The response option “No need for examination or treatment” was treated as missing value because it was not considered relevant when analyzing barriers to healthcare utilization. The presence of communicative barriers was assigned a value of 1 if at least one item was answered with “Yes.” The absence of communicative barriers was assigned a value of 0 if at least one item was answered with “No” and the other item was not answered with “Yes.” Cases were excluded if responses to both items were missing.

#### Financial barriers in healthcare utilization

Financial barriers in healthcare utilization were captured using the following two items: “Was there any time in the past 12 months when you needed the following kinds of health care, but could not afford it? (1) Medical care? (2) Prescribed medicines?” Response options were “Yes,” “No,” and “No need for [medical care/prescribed medicines].” The variable was likewise dichotomized for analysis. The response option “No need for [medical care/prescribed medicines]” was treated as missing. The presence of financial barriers was assigned a value of 1 if at least one item was answered with “Yes.” The absence of financial barriers was assigned a value of 0 if at least one item was answered with “No” and the other item was not answered with “Yes.” Cases were excluded if responses to both items were missing.

#### Demographic variables

Age was included as a continuous variable. Gender was categorized as male or female based on the self-reported gender entry on participants’ birth certificates. Information on sex assigned at birth and current gender identity was collected through self-report. Because of the small number of participants with diverse gender identities and due to a high number of missing values on gender identity, analyses beyond binary categories were not feasible. To avoid excluding participants and maintain statistical validity, the analyses were based on sex assigned at birth while acknowledging the limitations of this approach in capturing gender diversity.

#### Socioeconomic variables

Level of education was based on self-reported highest school and vocational qualifications and was categorized according to the International Standard Classification of Education (ISCED 2011) [30] into low (ISCED 1–2), medium (ISCED 3–4), and high (ISCED 5–8) education. Net equivalent income [31] was calculated using participants’ reports of monthly household net income and household composition (number of household members younger and older than 14 years). Participants were asked: “What is the total monthly net income of your household?”. If income information was missing, regression-based imputation methods were used, incorporating information on age, gender, employment status, household composition, education, occupational position, and regional data on unemployment and income tax [32]. Income groups were then categorized into low (quintile 1), medium (quintiles 2–4), and high (quintile 5) for the analysis. Subjective social status (SSS) was assessed using a German version of the MacArthur Scale of Subjective Social Status [33]. The following cutoffs were used: 1–4 = low SSS, 5–6 = medium SSS, and 7–10 = high SSS [33].

#### Migration-related variables

German language proficiency was assessed using two questions. Participants were asked about their native language and could indicate either “German” or “Another language.” Participants whose native language was not German were then asked: “How would you rate your German language proficiency?” Responses were recorded on a 5-point scale. For the present analysis, information from both questions was combined into the categories “native language/very good”, “good/average”, and “poor/very poor”. Duration of residence was operationalized using country of birth and year of immigration. Participants not born in Germany were asked the open-ended question: “When did you (first) move to Germany?” Duration of residence was categorized as “since birth,” “up to 10 years,” “11–30 years,” and “31 years or more.”

#### Psychosocial variables

Experiences of everyday discrimination were assessed using an adapted version of the Everyday Discrimination Scale [34, 35]. Participants who answered “Very often,” “Often,” “Sometimes,” or “Rarely” to at least one of the five situations presented were grouped together and compared with those who answered “Never” to all situations. Cases with more than two missing responses across the five items were excluded. Participants who reported experiences of discrimination were subsequently asked: “In your opinion, what was or were the reason(s) for these experiences?” [34]. Among the available response options, “origin, accent, language, appearance, name” was coded as the dichotomous variable “racist discrimination” (yes/no). The response options “level of education, income, occupation” and “unemployment” were coded as the dichotomous variable “discrimination due to socioeconomic situation” (yes/no).

In this analysis, only experiences of discrimination attributed to racism or the socioeconomic situation were considered.

### Data analysis

In the descriptive analyses, the prevalence of communicative and financial barriers was estimated with 95% confidence intervals (95% CIs) and stratified according to characteristics considered relevant for the multivariable analyses. Differences were considered statistically significant when the p-value from the chi-square test was < 0.05.

For the multivariable analyses, logistic regression models were calculated to identify associations between communicative and financial barriers and the predictor variables described above. Regression analyses were conducted separately for communicative and financial barriers because different variables may influence the occurrence of each type of barrier. Variable selection was guided by theoretical considerations. Odds ratios (OR) from univariable as well as adjusted OR (aOR) from multivariable regression models and corresponding 95% CIs are reported.

After setting the answer categories “No need for [examination/ treatment/ medical care/ prescribed medicines]” to missing, additionally for both outcome variables, participants with missing values in any of the relevant demographic, socioeconomic, migration-related, and psychosocial variables described above were excluded from the analyses (complete case analysis). This resulted in a sample size of 4,578 participants for the analyses of communicative barriers. For the analyses of financial barriers, the final sample consisted of 4,694 respondents. The differing sample sizes reflect the fact that the two outcomes were analyzed separately and included different variables.

A weighting factor was applied to adjust the sample to the population composition of the corresponding citizenship groups with respect to region, gender, age, education, and duration of residence [27]. Survey procedures for complex samples were used in all analyses to adequately account for clustering of participants within study locations and weighting in the calculation of confidence intervals and p-values.

The regression models were additionally adjusted for survey mode and citizenship according to residents’ registration offices to account for differences in response behavior [36]. All analyses were conducted using Stata/SE 17.0 [37].

## Results

### Sample description

Table 1 shows the distribution of demographic, socioeconomic, migration-related, and psychosocial characteristics within the study population. Slightly more than half of the participants were male, and the mean age was 42 years (range: 18–79 years). Nearly half of the participants belonged to the low educational group according to ISCED (45.6%). Nearly two-thirds of respondents (61.0%) were in the medium-income group, and most participants rated their subjective social status as medium. Only a minority of participants reported poor or very poor German language proficiency (7.3%). Approximately one-fifth of participants were born in Germany (19.8%), 27.8% held German citizenship according to self-report. Half of the participants reported racist discrimination (50.5%) and one in six participants (16.0%) reported experiences of discrimination related to their socioeconomic situation.

**Table 1:** Sample characteristics, GEDA Fokus, Germany, 2021–2022 (N = 6,038)

	**n**	**%**	**95** **% ** **CI**
**Demographic variables**			
**Gender**			
Male	2,983	46.2	43.5–49.0
Female	3,055	53.8	51.0–56.5
**Age**			
	6,038	41 (15.1)*	18–79*
**Socioeconomic** ** variables**			
**Educational level**			
Low	1,704	45.6	42.6–48.6
Medium	2,260	40.1	37.9–42.2
High	2,042	14.3	12.6–16.2
* missing*	32		
**Net equivalent income**			
Low	1,045	20.0	18.0–22.2
Medium	3,559	61.0	58.7–63.3
High	1,364	19.0	17.1–21.0
* missing*	70		
**Subjective social status**			
Low	1,920	33.2	30.0–36.3
Medium	2,266	41.5	38.6–44.4
High	1,597	25.3	23.1–27.6
* missing*	255		
**Migration-related variables**			
**German language proficiency**			
Native language / very good	2,570	45.3	42.0–48.6
Good / moderate	2,839	47.4	44.4–50.5
Poor / very poor	538	7.3	6.1–8.7
* missing*	91		
**Duration of residence**			
≤10 years	2,474	28.0	24.2–32.3
11–30 years	1,003	23.1	20.9–25.5
≥31 years	1,285	29.1	25.9–32.4
Since birth	1,189	19.8	17.6–22.3
* missing*	87		
**Psychosocial** ** variables**			
**Racist discrimination **			
Yes	3,047	50.0	46.9–53.0
No	2,830	50.0	47.0–53.1
* missing*	161		
**Discrimination due to socioeconomic situation**			
Yes	879	16.0	14.4–17.9
No	4,998	84.0	82.2–85.7
* missing*	161		
**Outcome variables**			
**Communicative barriers**			
Yes	838	17.4	
No	3,982	82.6	
No need for [examination /treatment] (*treated as missing*)	1153		
* missing*	65		
**Financial barriers**			
Yes	476	9.4	
No	4,571	90.6	
No need for [medical care /medicines] (*treated as missing*)	931		
* missing*	60		

n = unweighted, % weighted, CI = confidence interval

*Mean (standard deviation), range

### Communicative barriers in access to healthcare services

Of the 4,820 participants with valid information on communicative barriers, 4,570 participants were included in the analyses. Approximately one in six participants reported not having utilized healthcare services because of communicative barriers (16.2%; 95% CI: 14.4–18.2). According to the descriptive and univariable analyses (Table 2), communicative barriers were more common among females (19.7%), younger participants, participants with poor or very poor German language proficiency (31.5%), those with a duration of residence of up to 10 years (25.8%), and those who reported experiences of racist discrimination (21.9%).

The multivariable analyses largely confirmed these findings, except for duration of residence (Table 2). Female participants had higher odds of reporting communicative barriers than male participants (aOR 1.89; 95% CI: 1.40–2.56) and with every additional year of life, odds for reporting communicative barriers decreased (aOR 0.98; 95% CI: 0.97–0.99). Participants with poor or very poor German language proficiency had 2.5 times higher odds of reporting communicative barriers than those with native or very good German language proficiency (aOR 2.50; 95% CI: 1.65–3.80). Also, odds for those reporting good or moderate German language proficiency were increased (aOR 1.43; 95% CI: 1.01–1.99). In addition, experiences of racist discrimination were associated with higher odds of reporting communicative barriers (aOR 1.92; 95% CI: 1.29–2.87). Because the association between younger age and communicative barriers was unexpected, an interaction between age and duration of residence was examined (Supplementary tables S1, S2). The results indicated that communicative barriers were more common among younger individuals and those with a shorter duration of residence. Among individuals with a duration of residence of up to 10 years, the odds of not using healthcare services because of communicative barriers were 3.2 times higher (95% CI: 1.4–7.3; *p* = 0.005) for those aged 18–39 years, 2.9 times higher (95% CI: 1.2–6.8; *p* = 0.016) for those aged 40–59 years, and 3.6 times higher (95% CI: 1.1–12.0; *p* = 0.034) for those aged 60 years and older compared with individuals aged 40–59 years who had lived in Germany since birth. Individuals aged 18–39 years who had lived in Germany since birth had 2.5 times higher odds of not having utilized healthcare services because of communicative barriers than the reference group (95% CI: 1.1–5.8; *p* = 0.034).

**Table 2:** Descriptive, univariable, and multivariable analyses of communicative barriers to healthcare utilization (*N* = 4,578)

	Descriptive analysis							Univariable analysis					Multivariable analysis*				
	*n*_uw_	%_w_			95% CI	*p*-value		OR		95% CI	*p*-value		aOR		95% CI	*p*-value	
**Demographic variables**																	
**Gender**																	
Male (Ref.)	356/2,311		13.2	11.0–15.8			**0.002**	Ref.					Ref.				
Female	447/2,267		19.7	16.8–23.1				1.61	1.20–2.17			**0.002**	1.89	1.40–2.56			**< 0.001**
**Age**							**0.007**	0.98	0.97–0.99			**< 0.001**	0.98	0.97–0.99			**0.008**
**Socioeconomic variable**																	
**Educational level**																	
Low	212/1,290		15.9	13.1–19.1			0.906	0.95	0.71–1.29			0.757	1.15	0.82–1.63			0.416
Medium (Ref.)	323/1,725		16.5	14.1–19.2				Ref.					Ref.				
High	277/1,563		16.7	12.9–21.2				1.02	0.74–1.40			0.926	0.78	0.57–1.09			0.153
**Migration-related variables**																	
**German language proficiency**																	
Native language / very good (Ref.)	264/2,020		13.1	10.8–15.7			**< 0.001**	Ref.					Ref.				
Good / moderate	412/2,147		17.0	14.1–20.2				1.36	0.99–1.87			0.059	1.43	1.01–1.99			**0.038**
Poor / very poor	136/411		31.5	25.0–38.8				3.06	2.14–4.36			**< 0.001**	2.5	1.65–3.80			**< 0.001**
**Duration of residence**																	
Since birth (Ref.)	153/943		16.3	12.1–21.6			**< 0.001**	Ref.					Ref.				
≤ 10 years	471/1,893		25.8	22.0–29.9				1.79	1.20–2.66			**0.005**	1.62	0.97–2.70			0.063
11–30 years	92/774		10.3	7.6–13.8				0.59	0.35–0.99			**0.046**	0.66	0.36–1.22			0.182
≥ 31 years	96/968		11.8	8.9–15.3				0.69	0.43–1.10			0.118	0.90	0.47–1.72			0.743
**Psychosocial variable**																	
**Racist discrimination**																	
No (Ref.)	241/2,160		10.4	8.1–13.1			**< 0.001**	Ref.					Ref.				
Yes	571/2,418		21.9	19.0–25.0				2.42	1.71–3.43			**< 0.001**	1.92	1.29–2.87			**0.002**

n = unweighted count, % = weighted percentage, CI = confidence interval

*Adjusted for survey mode and citizenship according to residents’ registration offices

### Financial barriers in access to healthcare services

Of the 5,047 participants with valid information on financial barriers, 4,694 participants were included in the analyses. A total of 9.0% of participants (95% CI: 7.7–10.6) reported financial barriers to healthcare utilization. According to the descriptive and univariable analyses, financial barriers were more common among females (11.2%), participants with low subjective social status (12.4%), and respondents who reported experiences of discrimination related to their socioeconomic situation (18.7%). Financial barriers were less often reported by participants in the high income group (5.5%) (Table 3).

The multivariable analyses largely confirmed these findings, except for income and subjective social status. Female participants had higher odds of reporting financial barriers than male participants (aOR 1.80; 95% CI: 1.27–2.56). Participants who reported discrimination related to their socioeconomic situation also had higher odds of reporting financial barriers (aOR 2.75; 95% CI: 1.85–4.09). In contrast, a high educational level was associated with lower odds of reporting financial barriers compared to a medium educational level (aOR 0.63; 95% CI: 0.40–1.00; *p* = 0.050) (Table 3).

**Table 3:** Descriptive, univariable, and multivariable analyses of financial barriers to healthcare utilization (*N* = 4,694)

	Descriptive analysis						Univariable analysis			Multivariable analysis*		
	*n*_uw_	%_w_		95% CI	*p*-value		OR	95% CI	*p*-value	aOR	95% CI	*p*-value
**Demographic variables**												
**Gender**												
Male (Ref.)	186/2,351		7.1	5.5–9.1		**0.005**	Ref.			Ref.		
Female	260/2,351		11.2	9.1–13.8			1.66	1.16–2.37	**0.006**	1.80	1.27–2.56	**0.001**
**Age**						0.098	1.00	0.99–1.02	0.437	1.01	1.00–1.02	0.094
**Socioeconomic variables**												
**Educational level**												
Low	143/1,330		10.4	8.0–13.3		0.114	1.24	0.80–1.92	0.341	1.28	0.81–2.03	0.289
Medium (Ref.)	173/1,756		8.5	6.4–11.3			Ref.			Ref.		
High	130/1,608		6.0	4.6–7.9			0.69	0.45–1.05	0.081	0.63	0.40–1.00	**0.050**
**Net equivalent income**												
Low	105/818		9.1	6.6–12.4		**0.036**	0.89	0.59–1.34	0.580	0.77	0.51–1.16	0.210
Medium (Ref.)	291/2,804		10.1	8.1–12.5			Ref.			Ref.		
High	50/1,072		5.5	3.5–8.3			0.51	0.30–0.87	**0.015**	0.61	0.34–1.08	0.088
**Subjective social status**												
Low	216/1,597		12.4	9.4–16.0		**0.005**	1.88	1.22–2.89	**0.004**	1.55	0.98–2.25	0.062
Medium (Ref.)	126/1,843		7.0	5.3–9.2			Ref.			Ref.		
High	104/1,254		7.7	5.5–10.5			1.10	0.69–1.76	0.673	1.35	0.85–2.13	0.202
**Psychosocial variable**												
**Discrimination due to socioeconomic situation**												
No (Ref.)	317/3,981		7.1	5.8–8.7		**< 0.001**	Ref.			Ref.		
Yes	129/713		18.7	14.2–24.2			3.00	2.04–4.42	**< 0.001**	2.75	1.85–4.09	**< 0.001**

n = unweighted count, % = weighted percentage, CI = confidence interval

*Adjusted for survey mode and citizenship according to residents’ registration offices

## Discussion

Based on a sample of people with selected citizenships, this analysis shows that lower German language proficiency and experiences of racist discrimination are important factors associated with communicative barriers to accessing healthcare services. A high educational level was negatively associated with financial barriers, while female gender and experiences of discrimination due to socioeconomic circumstances were positively associated with financial barriers. According to the Federal Statistical Office, 26% (about 21 million people) of the population in Germany were immigrants or their descendants in 2024, defined as having either immigrated to Germany themselves or having two parents who immigrated to Germany since 1950. Among those, the biggest group is those who themselves or their parents were born in Turkiye (12%), followed by Poland (9%) [38]. However, figures according to the sampling characteristic of citizenship differ, but still the five selected citizenships are among the top ten citizenships recorded in the Central Register of Foreigners 2024 [39]. 

### Communicative barriers

One in six participants reported communicative barriers as a reason for not having utilized healthcare services. The results show that women reported not having utilized healthcare services because of communicative barriers more frequently than men. Previous studies indicate higher healthcare utilization among women, which has been linked to gynecological and obstetrical care, different health perceptions, a greater willingness to seek medical help, and caregiving responsibilities [16, 40–42]. Consequently, higher healthcare utilization may also be accompanied by more frequent reporting of barriers.

The results of this analysis show a significant association between age and communicative barriers, with the likelihood of reporting such barriers decreasing with each additional year of age. This finding contrasts with results from a German study indicating that language and information deficits represent central barriers to accessing the healthcare system, particularly among older migrants [43]. One possible explanation may lie in the relationship between age and duration of residence. In our sample, individuals with a shorter duration of residence, who were often younger, more frequently reported not having utilized healthcare services because of communicative barriers. A shorter duration of residence may reflect unfamiliarity with the healthcare system [44], whereas communicative barriers may decrease with increasing duration of residence. However, the higher odds observed among individuals aged 18–39 years who were born in Germany go along with the literature, as unmet needs overall are more often observed among younger individuals in Germany [45]. One study shows that navigational health literacy – generally low in Germany [46] – is lower among second generation migrants, which might explain our observation [45].

Educational level showed no significant association with communicative barriers. This finding is surprising, as previous studies have often reported a positive association between education and healthcare utilization, particularly specialist health care utilization [16, 19, 24]. However, such associations may also reflect the strong relationship between lower educational attainment, lower income, and poverty-related barriers to healthcare access [47]. The absence of a clear association in the present analysis may therefore be related to the heterogeneity of the sample and the interplay of multiple factors, including socioeconomic position and language proficiency, rather than educational level alone.

Findings from Germany [12] and international studies [10, 48] indicate that language proficiency is an important factor in explaining differences in healthcare utilization between migrants and non-migrants. In this study, individuals with German language proficiency lower than very good more frequently reported not having utilized healthcare services because of communicative barriers. This suggests that limited German language proficiency may hinder access to adequate healthcare [43]. Previous analyses of the GEDA Fokus study also found that self-assessed German language proficiency was associated with the utilization of general practitioner services [40]. Although duration of residence is often reported to be associated with healthcare utilization [22, 49], likely mediated through improved language proficiency [50], this association did not persist in the multivariable model.

To date, experiences of discrimination as a barrier to healthcare utilization have not been examined in such a differentiated manner with regard to self-reported reasons for discrimination. However, the general association between experiences of discrimination and access to healthcare has been described in the literature [11, 24, 42]. Hanssens et al. described the effects of discrimination in the healthcare system as twofold. First, discrimination interferes with equitable access to healthcare, and second, it is associated with lower levels of trust in healthcare professionals. This may result in delayed treatment [51]. Patients who experience discrimination tend to avoid contact with physicians or change physicians more frequently [19]. Treatment schedules may not be followed, and treatment may be discontinued altogether [17, 52].

### Financial barriers

Nearly one in ten participants reported financial barriers as a reason for not utilizing healthcare services. Such barriers may arise from mandatory co-payments, IGeL services, or a limited ability to cover out-of-pocket expenses. Gender appears to play a role in financial barriers to healthcare access. Women reported financial barriers more frequently than men. This finding may be linked to structural disadvantages in the labor market. On average, women earn less, are more often employed below their qualification level, and are disproportionately represented in precarious forms of employment [53]. The non-recognition of foreign qualifications, which affects both men and women, may further exacerbate these disadvantages [54]. These factors, combined with gendered role distributions that assign women a greater share of unpaid care work [55–58], increase the risk of poverty and reduce financial flexibility. In Germany, this may directly affect access to healthcare because statutory health insurance requires co-payments for services such as medication and hospital stays [3]. Consequently, women may experience financial barriers to healthcare more frequently, particularly when additional burdens such as single parenthood or caregiving responsibilities are present [59].

Furthermore, the analyses showed that people with a high educational level had lower odds of experiencing financial barriers when utilizing healthcare services. This may be related to greater access to financial resources. Individuals with higher educational attainment generally have better career prospects and, consequently, greater income opportunities and more favorable working conditions than those with lower educational attainment [60]. Improved career prospects and income opportunities may increase financial security and facilitate the utilization of healthcare services, particularly services that require out-of-pocket payments, such as IGeL services. In addition, higher educational attainment and the associated occupational opportunities may contribute to improved workplace health protection [61, 62], which may promote engagement with preventive health measures.

Previous research has shown that income is associated with healthcare utilization [63, 64]. Low income has been identified as a barrier to healthcare access among migrants [22]. In addition to direct costs for examinations and medication, indirect costs such as transportation, loss of earnings, and childcare may further limit access to healthcare services [22]. However, surprisingly in the analyses at hand income was not associated with the presence of financial barriers in the multivariable model.

Experiences of discrimination due to the socioeconomic situation played a significant role for reporting financial barriers in healthcare utilization. LaVeist et al. showed for Europe that income is among the most frequently cited reasons for perceived discrimination and that individuals with lower incomes report discrimination more often [65]. Hanssens et al. reported similar findings for the US [51] and also for Germany this can be observed [66]. Experiences of being devalued or treated less favorably because of one’s socioeconomic position may contribute to feelings of being undervalued and undermine trust in healthcare institution [67, 68].This may in turn reduce willingness to engage with healthcare services and make existing barriers to obtaining needed care more difficult to overcome. Thus, the observed relationship may reflect the interplay between material disadvantage and experiences of discrimination. 

### Strengths and limitations

Several limitations should be considered when interpreting the findings presented here. First, the cross-sectional design does not allow conclusions about the direction of the observed associations. Second, the barriers examined in this study were each assessed using only two items. It is therefore possible that some barriers to healthcare utilization were not captured. Furthermore, the questionnaire did not include a general question on barriers to healthcare utilization but instead asked about specific reasons for not utilizing healthcare services.

Additionally, the findings apply only to people with the selected citizenships included in the survey and cannot be generalized to all migrants and their descendants living in Germany. Nevertheless, these groups represent large migrant populations in Germany, and the study draws on the largest migration-related health survey conducted in the country to date, enabling in-depth analyses of the relationships between health status and migration-related, social, and structural determinants of health.

## Conclusion

For both communicative and financial barriers, our study identified important associations with female gender, for communicative barriers additionally with German language proficiency. Experiences of discrimination, whether racism-related or linked to the socioeconomic situation were relevant for both types of barriers examined. Experiences of discrimination may affect multiple dimensions of healthcare access, for example by undermining trust, limiting communication, and reinforcing social and economic exclusion. These findings suggest that barriers to healthcare utilization arise not only from individual circumstances but also from broader social determinants of health.

To make the healthcare system more inclusive and support more informed healthcare utilization among migrants, the broader use of qualified interpreters and digital language mediation tools could play an important role in reducing communicative barriers. Multilingual information materials may be particularly valuable for individuals who are not yet familiar with the German healthcare system. Discrimination needs to be addressed on a broader societal level as it constitutes a significant barrier to health care access and health equity, as also the World Health Organization emphasizes in their most recent World report on social determinants of health equity from 2025 [69]. In the healthcare sector itself, anti-discrimination and awareness-raising training for healthcare staff could foster respectful interactions and strengthen patient engagement. To address financial barriers, low-threshold and community-based services that offer voluntary access to diagnostics, primary care, and, where necessary, supplementary care may be beneficial. Strengthening public health services, taking into account the specific needs of women and families, and improving the physician–patient relationship through more time for consultations, targeted communication training, and reduced economic pressures may also contribute to improved healthcare access.

## Supplementary Information

Below is the link to the electronic supplementary material.

Supplementary Material 1

## Data Availability

The datasets generated during this study are not publicly available due to data protection requirements but are available from the corresponding author upon reasonable request.
