# Supplementary material for: Factors associated with communicative and financial barriers to healthcare utilization among people with selected citizenships in Germany: results from a nationwide cross-sectional study
Source: BMC Health Serv Res. 2026 Oct 24;26:1477. doi: 10.1186/s12913-026-15932-x (PMC13711910; doi:10.1186/s12913-026-15932-x)
Supplement: Supplementary file 1 [file 12913_2026_15932_MOESM1_ESM.docx]

**Supplementary Information**

**Factors associated with communicative and financial barriers to healthcare utilization among people with selected citizenships in Germany: Results from a nationwide cross-sectional study**

***Goerigk L, Kajikhina K, Hövener C, Koschollek C***

Table S1: Communicative barriers: Interaction between duration of residence and age group

| **Duration of Residence # Age Group** | **OR** | **95% CI** | **p-value** |
| --- | --- | --- | --- |
| ≤10 years # 18-39 years | 3.2 | 1.43–7.32 | **0.005** |
| ≤10 years # 40-59 years | 2.9 | 1.23–6.80 | **0.016** |
| ≤10 years # 60+ years | 3.6 | 1.10–12.00 | **0.034** |
| 11-30 years # 18-39 years | 1.8 | 0.64–4.79 | 0.272 |
| 11-30 years # 40-59 years | 0.8 | 0.31–2.12 | 0.672 |
| 11-30 years # 60+ years | 0.2 | 0.05–1.01 | 0.051 |
| ≥31 years # 18-39 years | 1.4 | 0.40–4.98 | 0.592 |
| ≥31 years # 40-59 years | 1.3 | 0.45–3.50 | 0.665 |
| ≥31 years # 60+ years | 0.9 | 0.37–2.44 | 0.907 |
| Since birth # 18-39 years | 2.5 | 1.07–5.82 | **0.034** |
| Since birth # 40-59 years | REF |  |  |
| Since brith # 60+ years | 0.1 | 0.01–1.35 | 0.086 |

Table S2: Communicative barriers, sensitivity analyses: Interaction between duration of residence and age group excluding those participants born in Germany

| **Duration of Residence # Age Group** | **OR** | **95% CI** | **p-value** |
| --- | --- | --- | --- |
| ≤10 years # 18-39 years | 2.5 | 1.26–4.92 | **0.009** |
| ≤10 years # 40-59 years | 2.3 | 1.05–5.01 | **0.037** |
| ≤10 years # 60+ years | 2.9 | 0.93–8.82 | 0.066 |
| 11-30 years # 18-39 years | 1.4 | 0.64–3.00 | 0.411 |
| 11-30 years # 40-59 years | 0.6 | 0.29–1.40 | 0.261 |
| 11-30 years # 60+ years | 0.2 | 0.04–0.78 | **0.023** |
| ≥31 years # 18-39 years | 1.1 | 0.32–3.97 | 0.857 |
| ≥31 years # 45-59 years | REF |  |  |
| ≥31 years # 60+ years | 0.8 | 0.37–1.65 | 0.510 |
